# *N*-[3-(5-Oxo-10,11-dihydro-5*H*-dibenzo[*a*,*d*]cyclo­hepten-2-ylamino)phen­yl]furan-3-carboxamide

**DOI:** 10.1107/S1600536810013450

**Published:** 2010-04-17

**Authors:** Angelika Dorn, Dieter Schollmeyer, Stefan A. Laufer

**Affiliations:** a Institute of Pharmacy, Department of Pharmaceutical and Medicinal Chemistry, Eberhard Karls University Tübingen, Auf der Morgenstelle 8, 72076 Tübingen, Germany; b Department of Organic Chemistry, ohannes Gutenberg-University Mainz, Duesbergweg 10-14, 55099 Mainz, Germany

## Abstract

In the title compound, C_26_H_20_N_2_O_3_, the two aromatic rings of the tricyclic unit are oriented at a dihedral angle of 54.53 (9)°. The crystal structure displays inter­molecular N—H⋯O hydrogen bonding.

## Related literature

For palladium-catalyzed amination reactions of aryl halides with anilines, see: Jensen *et al.* (2004 ▶); Grasa *et al.* (2001 ▶). For p38 inhibitors based on dibenzosuberones, see: Laufer *et al.* (2006 ▶). 
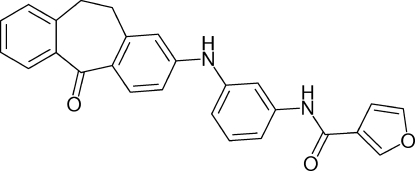


## Experimental

### 

#### Crystal data

C_26_H_20_N_2_O_3_
*M* *_r_* = 408.44
Monoclinic, 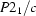

*a* = 10.7691 (7) Å
*b* = 21.746 (1) Å
*c* = 8.8666 (6) Å
β = 101.934 (2)°
*V* = 2031.6 (2) Å^3^
*Z* = 4
Mo *K*α radiation
μ = 0.09 mm^−1^
*T* = 173 K
0.30 × 0.20 × 0.10 mm

#### Data collection

Bruker SMART APEXII diffractometer
22214 measured reflections
4856 independent reflections
3347 reflections with *I* > 2σ(*I*)
*R*_int_ = 0.044

#### Refinement

*R*[*F* ^2^ > 2σ(*F* ^2^)] = 0.050
*wR*(*F* ^2^) = 0.115
*S* = 0.99
4856 reflections
280 parameters
H-atom parameters constrained
Δρ_max_ = 0.24 e Å^−3^
Δρ_min_ = −0.30 e Å^−3^

### 

Data collection: *APEX2* (Bruker, 2006 ▶); cell refinement: *SAINT* (Bruker, 2006 ▶); data reduction: *SAINT*; program(s) used to solve structure: *SIR97* (Altomare *et al.*, 1999 ▶); program(s) used to refine structure: *SHELXL97* (Sheldrick, 2008 ▶); molecular graphics: *PLATON* (Spek, 2009 ▶); software used to prepare material for publication: *PLATON*.

## Supplementary Material

Crystal structure: contains datablocks I, global. DOI: 10.1107/S1600536810013450/im2187sup1.cif

Structure factors: contains datablocks I. DOI: 10.1107/S1600536810013450/im2187Isup2.hkl

Additional supplementary materials:  crystallographic information; 3D view; checkCIF report

## Figures and Tables

**Table 1:** Hydrogen-bond geometry (Å, °)

*D*—H⋯*A*	*D*—H	H⋯*A*	*D*⋯*A*	*D*—H⋯*A*
N17—H17⋯O16^i^	0.87	2.14	2.900 (2)	146
N24—H24⋯O26^ii^	0.91	2.00	2.839 (2)	153

Symmetry codes: (i) 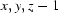; (ii) 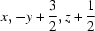.

**Fig. 1.**
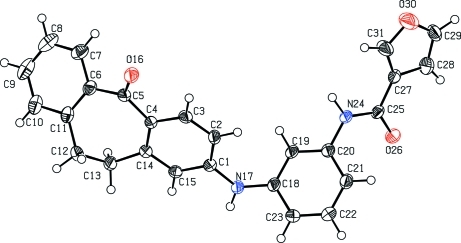
View of compound I. Displacement ellipsoids are drawn at the 50% probability level.

**Fig. 2.**
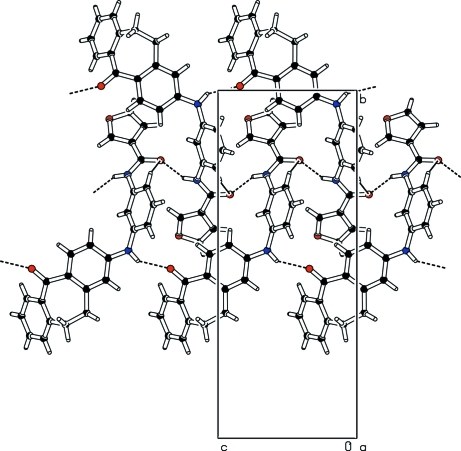
Part of the crystal packing showing the supramoleculare structure. View along the a-axis.

## supplementary crystallographic information

### Comment

p38 mitogen activated protein (MAP) kinase is a key enzyme in inflammatory diseases as it is involved in the biosynthesis of proinflammatory cytokines such as TNF-α and IL-1β (Laufer *et al.* 2006). Small molecule p38 inhibitors suppress the production of these cytokines and therefore p38 is an attractive and promising drug target for novel anti-inflammatory therapeutics (Laufer *et al.* 2006). Recently, we designed and synthesized a series of p38 inhibitors based on dibenzosuberones (Laufer *et al.* 2006). The title compound was prepared in the course of our studies on dibenzo[a,d]cycloheptan-5-ones as potent p38 MAP kinase inhibitors.

The structure of the title compound, at 173 (2) K has monoclinic (*P*2_1_/*c*) symmetry. In the molecule (Fig.1), rings A (C1—C4, C14, C15) and B (C6—C11) are, of course, planar and they are oriented at a dihedral angle of A/B = 54.53 (9)°. The intramolecular C21—H21···O26 (2.66 Å) interaction stabilizes the conformation of the molecule. In the crystal structure the hydrogen bonds N17—H17···O16 (2.90 Å) and N24—H24···O26 (2.84 Å) link the molecule into double layers.

### Experimental

For the preparation of the title compound a mixture of 500 mg (2.1 mmol) 2-chloro-10,11-dihydro-5*H*-dibenzo[*a,d*][7]annulen-5-one, 420 mg (2.1 mmol) *N*-(3-aminophenyl)-3-furamide, 940 mg (8.4 mmol) KO*tert*-Bu, 90 mg (0.19 mmol) 2-(dicyclohexylphosphino)-2'-, 4'-, 6'-triisopropylbiphenyl and 20 mg (0.09 mmol) Pd(OAc)_2_ in 3 ml absolute *tert-*butanol and 7 ml absolute toluol was stirred for 4 h at 363 K under an atmosphere of argon. The mixture was diluted with water and then extracted with ethyl acetate. The extracts were combined, washed with saturated saline solution, dried over Na_2_SO_4_ and then evaporated under reduced pressure. The residue was purified by flash chromatography (SiO_2_ 60, *n-*hexane / ethyl acetate 3 + 2) (yield: 17.2 %). Crystals of the title compound were obtained by slow evaporation of a methanol / diethyl ether solution at room temperature.

### Refinement

Hydrogen atoms attached to carbons were placed at calculated positions with C—H = 0.95 Å (aromatic) or 0.98–0.99 Å (*sp*^3^ C-atom). Hydrogen atoms attached to N17 and N24 were located in diff. Fourier maps. All H atoms were refined in the riding-model approximation with isotropic displacement parameters (set at 1.2–1.5 times of the *U*_eq_ of the parent atom).

### Crystal data

**Table:**

C_26_H_20_N_2_O_3_	*F*(000) = 856
*M**_r_* = 408.44	*D*_x_ = 1.335 Mg m^−^^3^
Monoclinic, *P*2_1_/*c*	Mo *K*α radiation, λ = 0.71069 Å
Hall symbol: -P 2ybc	Cell parameters from 3910 reflections
*a* = 10.7691 (7) Å	θ = 2.5–25.8°
*b* = 21.746 (1) Å	µ = 0.09 mm^−^^1^
*c* = 8.8666 (6) Å	*T* = 173 K
β = 101.934 (2)°	Plate, yellow
*V* = 2031.6 (2) Å^3^	0.30 × 0.20 × 0.10 mm
*Z* = 4	

### Data collection

**Table:**

Bruker SMART APEXII diffractometer	3347 reflections with *I* > 2σ(*I*)
Radiation source: sealed Tube	*R*_int_ = 0.044
graphite	θ_max_ = 27.9°, θ_min_ = 1.9°
CCD scan	*h* = −14→14
22214 measured reflections	*k* = −28→28
4856 independent reflections	*l* = −11→11

### Refinement

**Table:**

Refinement on *F*^2^	Primary atom site location: structure-invariant direct methods
Least-squares matrix: full	Secondary atom site location: difference Fourier map
*R*[*F*^2^ > 2σ(*F*^2^)] = 0.050	Hydrogen site location: inferred from neighbouring sites
*wR*(*F*^2^) = 0.115	H-atom parameters constrained
*S* = 0.99	*w* = 1/[σ^2^(*F*_o_^2^) + (0.0358*P*)^2^ + 1.2737*P*] where *P* = (*F*_o_^2^ + 2*F*_c_^2^)/3
4856 reflections	(Δ/σ)_max_ < 0.001
280 parameters	Δρ_max_ = 0.24 e Å^−^^3^
0 restraints	Δρ_min_ = −0.30 e Å^−^^3^

### Special details

**Table:**

Geometry. All esds (except the esd in the dihedral angle between two l.s. planes) are estimated using the full covariance matrix. The cell esds are taken into account individually in the estimation of esds in distances, angles and torsion angles; correlations between esds in cell parameters are only used when they are defined by crystal symmetry. An approximate (isotropic) treatment of cell esds is used for estimating esds involving l.s. planes.
Refinement. Refinement of *F*^2^ against ALL reflections. The weighted *R*-factor wR and goodness of fit *S* are based on *F*^2^, conventional *R*-factors *R* are based on *F*, with *F* set to zero for negative *F*^2^. The threshold expression of *F*^2^ > σ(*F*^2^) is used only for calculating *R*-factors(gt) etc. and is not relevant to the choice of reflections for refinement. *R*-factors based on *F*^2^ are statistically about twice as large as those based on *F*, and *R*- factors based on ALL data will be even larger.

### Fractional atomic coordinates and isotropic or equivalent isotropic displacement parameters (Å^2^)

**Table:**

	*x*	*y*	*z*	*U*_iso_*/*U*_eq_	
C1	0.33786 (17)	0.51791 (8)	0.78886 (19)	0.0268 (4)	
C2	0.35431 (17)	0.55527 (8)	0.92028 (19)	0.0282 (4)	
H2	0.3714	0.5979	0.9131	0.034*	
C3	0.34553 (17)	0.52992 (8)	1.05935 (19)	0.0278 (4)	
H3	0.3579	0.5559	1.1474	0.033*	
C4	0.31908 (16)	0.46745 (8)	1.07779 (18)	0.0255 (4)	
C5	0.30193 (18)	0.45074 (8)	1.23480 (19)	0.0293 (4)	
C6	0.23344 (17)	0.39443 (8)	1.26987 (19)	0.0306 (4)	
C7	0.15301 (19)	0.40150 (10)	1.3739 (2)	0.0394 (5)	
H7	0.1435	0.4409	1.4166	0.047*	
C8	0.0873 (2)	0.35182 (12)	1.4151 (3)	0.0521 (6)	
H8	0.0319	0.3572	1.4847	0.063*	
C9	0.1023 (2)	0.29455 (11)	1.3551 (3)	0.0528 (6)	
H9	0.0572	0.2603	1.3834	0.063*	
C10	0.1828 (2)	0.28669 (10)	1.2539 (2)	0.0420 (5)	
H10	0.1932	0.2469	1.2141	0.050*	
C11	0.24914 (18)	0.33624 (8)	1.2092 (2)	0.0323 (4)	
C12	0.3358 (2)	0.32699 (8)	1.0986 (2)	0.0359 (4)	
H12A	0.3430	0.2825	1.0788	0.043*	
H12B	0.4215	0.3425	1.1458	0.043*	
C13	0.2882 (2)	0.36007 (8)	0.9458 (2)	0.0345 (4)	
H13A	0.3316	0.3422	0.8680	0.041*	
H13B	0.1964	0.3514	0.9118	0.041*	
C14	0.30687 (17)	0.42898 (8)	0.94687 (19)	0.0269 (4)	
C15	0.31629 (17)	0.45534 (8)	0.80680 (19)	0.0272 (4)	
H15	0.3076	0.4294	0.7189	0.033*	
O16	0.33648 (14)	0.48724 (6)	1.34167 (14)	0.0397 (3)	
N17	0.34715 (16)	0.53956 (6)	0.64489 (16)	0.0325 (4)	
H17	0.3530	0.5103	0.5792	0.039*	
C18	0.36701 (17)	0.60072 (8)	0.60389 (18)	0.0279 (4)	
C19	0.29900 (17)	0.64925 (8)	0.65027 (18)	0.0269 (4)	
H19	0.2392	0.6415	0.7131	0.032*	
C20	0.31876 (17)	0.70897 (8)	0.60449 (18)	0.0267 (4)	
C21	0.40671 (18)	0.72084 (8)	0.5136 (2)	0.0312 (4)	
H21	0.4230	0.7619	0.4863	0.037*	
C22	0.47030 (18)	0.67210 (9)	0.4632 (2)	0.0344 (4)	
H22	0.5280	0.6798	0.3976	0.041*	
C23	0.45104 (18)	0.61242 (8)	0.5069 (2)	0.0321 (4)	
H23	0.4950	0.5794	0.4709	0.039*	
N24	0.24633 (15)	0.75736 (6)	0.65095 (15)	0.0291 (3)	
H24	0.2255	0.7522	0.7444	0.035*	
C25	0.18520 (17)	0.80036 (8)	0.55246 (18)	0.0262 (4)	
O26	0.18260 (14)	0.79914 (6)	0.41345 (14)	0.0400 (3)	
C27	0.12282 (16)	0.85057 (8)	0.61918 (19)	0.0265 (4)	
C28	0.04935 (19)	0.89762 (9)	0.5342 (2)	0.0392 (5)	
H28	0.0280	0.9008	0.4250	0.047*	
C29	0.01532 (18)	0.93667 (9)	0.6332 (2)	0.0373 (4)	
H29	−0.0351	0.9724	0.6059	0.045*	
O30	0.06443 (17)	0.91741 (7)	0.78210 (18)	0.0579 (4)	
C31	0.1293 (2)	0.86527 (9)	0.7692 (2)	0.0451 (5)	
H31	0.1737	0.8419	0.8540	0.054*	

### Atomic displacement parameters (Å^2^)

**Table:**

	*U*^11^	*U*^22^	*U*^33^	*U*^12^	*U*^13^	*U*^23^
C1	0.0327 (9)	0.0268 (9)	0.0223 (8)	0.0032 (7)	0.0090 (7)	0.0015 (7)
C2	0.0391 (10)	0.0206 (8)	0.0256 (8)	−0.0007 (7)	0.0085 (7)	0.0008 (7)
C3	0.0372 (10)	0.0252 (9)	0.0212 (8)	0.0037 (7)	0.0066 (7)	−0.0032 (7)
C4	0.0328 (9)	0.0227 (8)	0.0222 (8)	0.0043 (7)	0.0087 (7)	0.0015 (6)
C5	0.0394 (10)	0.0263 (9)	0.0238 (8)	0.0076 (8)	0.0103 (7)	0.0019 (7)
C6	0.0350 (10)	0.0349 (10)	0.0222 (8)	0.0031 (8)	0.0062 (7)	0.0055 (7)
C7	0.0417 (11)	0.0512 (12)	0.0266 (9)	0.0012 (9)	0.0099 (8)	0.0027 (8)
C8	0.0502 (13)	0.0741 (17)	0.0365 (11)	−0.0102 (12)	0.0191 (10)	0.0075 (11)
C9	0.0576 (14)	0.0616 (15)	0.0399 (12)	−0.0216 (12)	0.0118 (10)	0.0123 (11)
C10	0.0523 (13)	0.0376 (11)	0.0346 (10)	−0.0071 (9)	0.0056 (9)	0.0092 (8)
C11	0.0367 (10)	0.0314 (10)	0.0277 (9)	0.0010 (8)	0.0042 (8)	0.0079 (7)
C12	0.0489 (12)	0.0223 (9)	0.0389 (10)	0.0044 (8)	0.0146 (9)	0.0030 (8)
C13	0.0557 (12)	0.0220 (9)	0.0299 (9)	−0.0011 (8)	0.0186 (9)	−0.0022 (7)
C14	0.0319 (9)	0.0230 (8)	0.0274 (9)	0.0016 (7)	0.0099 (7)	0.0002 (7)
C15	0.0372 (10)	0.0232 (9)	0.0227 (8)	0.0004 (7)	0.0095 (7)	−0.0048 (7)
O16	0.0678 (10)	0.0308 (7)	0.0232 (6)	0.0006 (7)	0.0153 (6)	−0.0017 (5)
N17	0.0580 (10)	0.0222 (7)	0.0210 (7)	0.0026 (7)	0.0165 (7)	−0.0009 (6)
C18	0.0394 (10)	0.0263 (9)	0.0184 (8)	0.0003 (8)	0.0072 (7)	0.0013 (7)
C19	0.0374 (10)	0.0285 (9)	0.0161 (7)	−0.0002 (7)	0.0083 (7)	0.0009 (6)
C20	0.0358 (10)	0.0267 (9)	0.0170 (7)	0.0007 (7)	0.0042 (7)	−0.0009 (6)
C21	0.0388 (10)	0.0268 (9)	0.0293 (9)	−0.0033 (8)	0.0097 (8)	0.0021 (7)
C22	0.0375 (10)	0.0358 (10)	0.0337 (10)	0.0002 (8)	0.0163 (8)	0.0032 (8)
C23	0.0400 (10)	0.0297 (9)	0.0294 (9)	0.0051 (8)	0.0137 (8)	−0.0007 (7)
N24	0.0469 (9)	0.0267 (8)	0.0162 (7)	0.0040 (7)	0.0123 (6)	0.0019 (6)
C25	0.0347 (9)	0.0259 (9)	0.0191 (8)	−0.0048 (7)	0.0083 (7)	0.0001 (7)
O26	0.0628 (9)	0.0406 (8)	0.0188 (6)	0.0125 (7)	0.0136 (6)	0.0047 (5)
C27	0.0316 (9)	0.0249 (9)	0.0236 (8)	−0.0032 (7)	0.0070 (7)	0.0018 (7)
C28	0.0436 (11)	0.0396 (11)	0.0331 (10)	0.0046 (9)	0.0046 (9)	0.0084 (8)
C29	0.0349 (10)	0.0269 (10)	0.0503 (12)	0.0082 (8)	0.0093 (9)	0.0054 (8)
O30	0.0789 (12)	0.0513 (10)	0.0456 (9)	0.0208 (9)	0.0179 (8)	−0.0055 (7)
C31	0.0696 (15)	0.0386 (11)	0.0267 (10)	0.0212 (11)	0.0087 (9)	−0.0009 (8)

### Geometric parameters (Å, °)

**Table:**

C1—N17	1.384 (2)	C14—C15	1.391 (2)
C1—C15	1.395 (2)	C15—H15	0.9500
C1—C2	1.401 (2)	N17—C18	1.407 (2)
C2—C3	1.372 (2)	N17—H17	0.8739
C2—H2	0.9500	C18—C19	1.394 (2)
C3—C4	1.405 (2)	C18—C23	1.395 (2)
C3—H3	0.9500	C19—C20	1.390 (2)
C4—C14	1.415 (2)	C19—H19	0.9500
C4—C5	1.487 (2)	C20—C21	1.389 (2)
C5—O16	1.233 (2)	C20—N24	1.421 (2)
C5—C6	1.495 (3)	C21—C22	1.385 (3)
C6—C7	1.398 (3)	C21—H21	0.9500
C6—C11	1.399 (3)	C22—C23	1.382 (3)
C7—C8	1.382 (3)	C22—H22	0.9500
C7—H7	0.9500	C23—H23	0.9500
C8—C9	1.377 (3)	N24—C25	1.354 (2)
C8—H8	0.9500	N24—H24	0.9089
C9—C10	1.381 (3)	C25—O26	1.2274 (19)
C9—H9	0.9500	C25—C27	1.469 (2)
C10—C11	1.395 (3)	C27—C31	1.356 (2)
C10—H10	0.9500	C27—C28	1.412 (2)
C11—C12	1.501 (3)	C28—C29	1.326 (3)
C12—C13	1.525 (3)	C28—H28	0.9500
C12—H12A	0.9900	C29—O30	1.382 (2)
C12—H12B	0.9900	C29—H29	0.9500
C13—C14	1.512 (2)	O30—C31	1.349 (2)
C13—H13A	0.9900	C31—H31	0.9500
C13—H13B	0.9900		
			
N17—C1—C15	118.86 (15)	C15—C14—C13	115.93 (15)
N17—C1—C2	123.30 (16)	C4—C14—C13	125.51 (15)
C15—C1—C2	117.79 (15)	C14—C15—C1	123.27 (15)
C3—C2—C1	119.60 (16)	C14—C15—H15	118.4
C3—C2—H2	120.2	C1—C15—H15	118.4
C1—C2—H2	120.2	C1—N17—C18	127.17 (14)
C2—C3—C4	123.15 (16)	C1—N17—H17	113.4
C2—C3—H3	118.4	C18—N17—H17	118.7
C4—C3—H3	118.4	C19—C18—C23	119.41 (16)
C3—C4—C14	117.59 (15)	C19—C18—N17	121.48 (15)
C3—C4—C5	114.38 (14)	C23—C18—N17	118.99 (15)
C14—C4—C5	127.98 (15)	C20—C19—C18	119.97 (16)
O16—C5—C4	119.09 (16)	C20—C19—H19	120.0
O16—C5—C6	116.80 (15)	C18—C19—H19	120.0
C4—C5—C6	123.85 (15)	C21—C20—C19	120.47 (16)
C7—C6—C11	119.61 (17)	C21—C20—N24	120.79 (15)
C7—C6—C5	116.52 (17)	C19—C20—N24	118.74 (15)
C11—C6—C5	123.83 (16)	C22—C21—C20	119.14 (16)
C8—C7—C6	120.6 (2)	C22—C21—H21	120.4
C8—C7—H7	119.7	C20—C21—H21	120.4
C6—C7—H7	119.7	C23—C22—C21	121.02 (17)
C9—C8—C7	119.8 (2)	C23—C22—H22	119.5
C9—C8—H8	120.1	C21—C22—H22	119.5
C7—C8—H8	120.1	C22—C23—C18	119.89 (16)
C8—C9—C10	120.2 (2)	C22—C23—H23	120.1
C8—C9—H9	119.9	C18—C23—H23	120.1
C10—C9—H9	119.9	C25—N24—C20	123.20 (14)
C9—C10—C11	121.1 (2)	C25—N24—H24	119.6
C9—C10—H10	119.5	C20—N24—H24	115.3
C11—C10—H10	119.5	O26—C25—N24	122.68 (16)
C10—C11—C6	118.66 (18)	O26—C25—C27	120.20 (16)
C10—C11—C12	120.43 (17)	N24—C25—C27	117.10 (14)
C6—C11—C12	120.91 (16)	C31—C27—C28	105.22 (16)
C11—C12—C13	112.11 (16)	C31—C27—C25	129.44 (16)
C11—C12—H12A	109.2	C28—C27—C25	125.17 (16)
C13—C12—H12A	109.2	C29—C28—C27	108.14 (17)
C11—C12—H12B	109.2	C29—C28—H28	125.9
C13—C12—H12B	109.2	C27—C28—H28	125.9
H12A—C12—H12B	107.9	C28—C29—O30	109.57 (17)
C14—C13—C12	116.24 (16)	C28—C29—H29	125.2
C14—C13—H13A	108.2	O30—C29—H29	125.2
C12—C13—H13A	108.2	C31—O30—C29	106.05 (15)
C14—C13—H13B	108.2	O30—C31—C27	111.01 (17)
C12—C13—H13B	108.2	O30—C31—H31	124.5
H13A—C13—H13B	107.4	C27—C31—H31	124.5
C15—C14—C4	118.53 (15)		
			
N17—C1—C2—C3	−178.94 (17)	C4—C14—C15—C1	0.3 (3)
C15—C1—C2—C3	−1.7 (3)	C13—C14—C15—C1	−177.81 (17)
C1—C2—C3—C4	−0.7 (3)	N17—C1—C15—C14	179.27 (17)
C2—C3—C4—C14	2.9 (3)	C2—C1—C15—C14	1.9 (3)
C2—C3—C4—C5	−174.59 (17)	C15—C1—N17—C18	179.19 (17)
C3—C4—C5—O16	−14.7 (2)	C2—C1—N17—C18	−3.6 (3)
C14—C4—C5—O16	168.12 (18)	C1—N17—C18—C19	−45.9 (3)
C3—C4—C5—C6	159.21 (16)	C1—N17—C18—C23	138.03 (19)
C14—C4—C5—C6	−18.0 (3)	C23—C18—C19—C20	−2.4 (3)
O16—C5—C6—C7	35.6 (2)	N17—C18—C19—C20	−178.41 (16)
C4—C5—C6—C7	−138.42 (18)	C18—C19—C20—C21	−0.5 (3)
O16—C5—C6—C11	−142.16 (18)	C18—C19—C20—N24	178.77 (15)
C4—C5—C6—C11	43.8 (3)	C19—C20—C21—C22	2.9 (3)
C11—C6—C7—C8	−1.1 (3)	N24—C20—C21—C22	−176.33 (16)
C5—C6—C7—C8	−178.99 (18)	C20—C21—C22—C23	−2.5 (3)
C6—C7—C8—C9	0.9 (3)	C21—C22—C23—C18	−0.4 (3)
C7—C8—C9—C10	0.0 (3)	C19—C18—C23—C22	2.8 (3)
C8—C9—C10—C11	−0.7 (3)	N17—C18—C23—C22	178.94 (17)
C9—C10—C11—C6	0.5 (3)	C21—C20—N24—C25	47.7 (2)
C9—C10—C11—C12	−179.72 (19)	C19—C20—N24—C25	−131.59 (18)
C7—C6—C11—C10	0.4 (3)	C20—N24—C25—O26	3.6 (3)
C5—C6—C11—C10	178.11 (17)	C20—N24—C25—C27	−175.05 (15)
C7—C6—C11—C12	−179.36 (17)	O26—C25—C27—C31	−169.7 (2)
C5—C6—C11—C12	−1.7 (3)	N24—C25—C27—C31	9.0 (3)
C10—C11—C12—C13	114.25 (19)	O26—C25—C27—C28	4.7 (3)
C6—C11—C12—C13	−66.0 (2)	N24—C25—C27—C28	−176.57 (17)
C11—C12—C13—C14	75.8 (2)	C31—C27—C28—C29	−0.6 (2)
C3—C4—C14—C15	−2.7 (2)	C25—C27—C28—C29	−176.09 (17)
C5—C4—C14—C15	174.47 (17)	C27—C28—C29—O30	0.4 (2)
C3—C4—C14—C13	175.28 (17)	C28—C29—O30—C31	0.0 (2)
C5—C4—C14—C13	−7.6 (3)	C29—O30—C31—C27	−0.3 (3)
C12—C13—C14—C15	153.30 (17)	C28—C27—C31—O30	0.6 (2)
C12—C13—C14—C4	−24.7 (3)	C25—C27—C31—O30	175.83 (18)

### Hydrogen-bond geometry (Å, °)

**Table:**

*D*—H···*A*	*D*—H	H···*A*	*D*···*A*	*D*—H···*A*
N17—H17···O16^i^	0.87	2.14	2.900 (2)	146
N24—H24···O26^ii^	0.91	2.00	2.839 (2)	153
C21—H21···O21	0.95	2.66	2.937 (2)	97

Symmetry codes: (i) *x*, *y*, *z*−1; (ii) *x*, −*y*+3/2, *z*+1/2.
